# No Effect of a Single Session of Transcranial Direct Current Stimulation on Experimentally Induced Pain in Patients with Chronic Low Back Pain – An Exploratory Study

**DOI:** 10.1371/journal.pone.0048857

**Published:** 2012-11-26

**Authors:** Kerstin Luedtke, Arne May, Tim P. Jürgens

**Affiliations:** Department of Systems Neuroscience, University Medical Centre Hamburg-Eppendorf, Hamburg, Germany; The James Cook University Hospital, United Kingdom

## Abstract

Transcranial direct current stimulation (tDCS) has been shown to modulate cortical excitability. A small number of studies suggested that tDCS modulates the response to experimental pain paradigms. No trials have been conducted to evaluate the response of patients already suffering from pain, to an additional experimental pain before and after tDCS. The present study investigated the effect of a single session of anodal, cathodal and sham stimulation (15 mins/1 mA) over the primary motor cortex on the perceived intensity of repeated noxious thermal and electrical stimuli and on elements of quantitative sensory testing (thermal pain and perception thresholds) applied to the right hand in 15 patients with chronic low back pain. The study was conducted in a double-blind sham-controlled and cross-over design. No significant alterations of pain ratings were found. Modalities of quantitative sensory testing remained equally unchanged. It is therefore hypothesized that a single 15 mins session of tDCS at 1 mA may not be sufficient to alter the perception of experimental pain and in patients with chronic pain. Further studies applying repetitive tDCS to patients with chronic pain are required to fully answer the question whether experimental pain perception may be influenced by tDCS over the motor cortex.

## Introduction

Transcranial direct current stimulation (tDCS) is a non-invasive brain stimulation technique that modulates the neuronal resting membrane potential and thereby affects neuronal activity [1]. Direct current stimulation of the motor cortex leads to local and referred excitability changes [2]. Cortical excitability increases during anodal stimulation and is reduced during cathodal stimulation as confirmed by changes of magneto-electric evoked potentials [3] and activation of the motor cortex in functional magnetic resonance imaging (fMRI) [4].

A pain reducing effect of tDCS on different types of experimental pain has been reported by a number of studies in healthy participants only [5], [6], [7], [8], [9]. However, the evidence is inconsistent regarding the most effective stimulation approach in terms of stimulation site and polarity. Some studies reported analgesic effects from cathodal stimulation, both over the somatosensory cortex (S1) [5] and over the motor cortex [7]. In contrast, Boggio and colleagues reported beneficial effects of tDCS on the perceived intensity of an electrical pain paradigm and an increase of perception thresholds after anodal stimulation over the motor cortex [6]. Recently our workgroup conducted a clinical trial that attempted to replicate these effects but found that experimental pain as well as quantitative sensory testing results remained unaffected by a single session of anodal tDCS to the motor cortex in healthy participants [10].

Stimulation paradigms chosen for studies on the reduction of chronic clinical pain were more consistent: all authors applied anodal tDCS over the motor cortex. A significant pain reduction was reported in patients with fibromyalgia [11], spinal cord injury [12], multiple sclerosis [13], chronic pelvic pain [14] and various other chronic pain conditions [15], [16]. This was supported by a systematic review and meta-analysis that found a superiority of active over sham tDCS in a subgroup analysis of tDCS applied to the motor cortex, the most frequently targeted stimulation site. However, the combined effect for pain reduction was based on only 5 studies with small sample sizes and large confidence intervals, not allowing for firm conclusions regarding the efficacy of tDCS [17].

Moreover, the neurophysiological background has still not been fully understood and little is known about the effects of tDCS on experimental pain in patients with chronic pain. As chronic noxious input causes widespread structural changes to the brain [18], [19], [20] and extensive functional changes to the nociceptive system [21], [22], [23], [24], it is tempting to speculate that effects of tDCS could be more pronounced in patients with chronic pain compared to healthy participants. However, to date this has only been examined in repetitive transcranial magnetic brain stimulation (rTMS) of the motor cortex: a recent study has shown an attenuation of experimental hand pain in chronic neuropathic pain [25]. Another study on patients with chronic back pain demonstrated a modulation of sensory thresholds after rTMS measured at the contralateral hand [26].

In conclusion, it is unknown at present whether

tDCS alters the perceived pain intensity of an experimental pain paradigm in patients with chronic pain.
anodal or cathodal tDCS is more effective to influence experimental pain in patients with chronic pain.
tDCS influences both thermal and electrical pain in the same way.

Consequently, this study investigates whether tDCS reduces the perceived intensity of experimental pain (thermal and electrical) and modalities of quantitative sensory testing (thermal pain and perception thresholds) in patients with chronic low back pain.

## Methods

### Study design

Fifteen patients with chronic low back pain were included into a double-blind sham-controlled crossover trial to study the effects of anodal, cathodal and sham tDCS on experimental thermal and electrical pain.

### Study Participants

Eligible patients were recruited among participants of a pain management group program for low back pain at a specialized clinic in Germany. Inclusion criteria were: 18 years or older and a minimum of 6 months of low back pain, defined according to the “European Guidelines of Chronic Low Back Pain” [27]. Exclusion criteria were immovable metal objects in and around the head, pregnancy, psychiatric disease (such as major depression, schizophrenia), neurologic (such as epilepsy, Parkinson's disease, stroke) or severe other diseases (such as diabetes, rheumatoid arthritis). If prophylactic medication was used, intake was recorded and had to remain stable throughout the study period. Participation was voluntary. All subjects provided written informed consent prior to inclusion into the study. The study was approved by the local Ethics Committee and conformed to the Declaration of Helsinki (World Medical Association 2008).

### Randomization procedure

**Figure 1:**
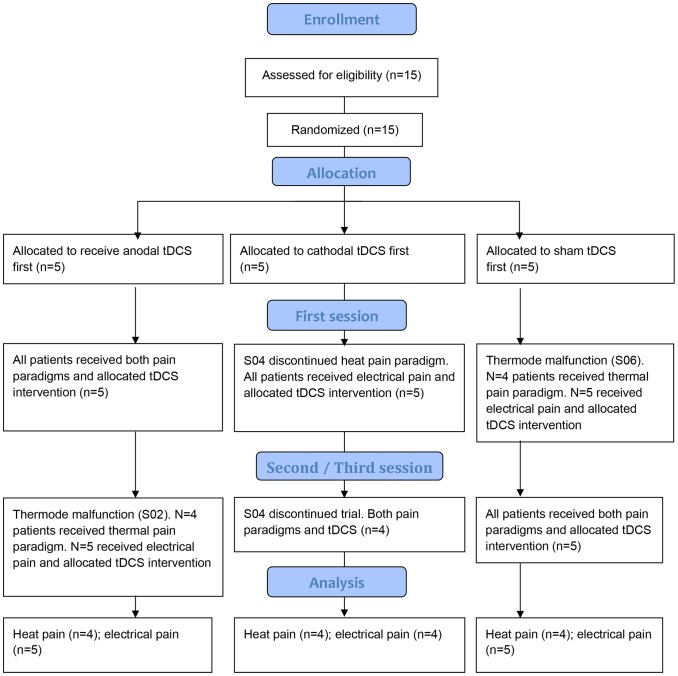
Flowchart of patient inclusion (CONSORT 2010).

Each participant received all three modes of stimulation (anodal, cathodal, and sham). The order of stimulation polarity was randomized according to a computer generated randomization list that matched study entry numbers with stimulation codes (see Figure 1 with flowchart for further details on patient inclusion).

### Measurements

**Figure 2:**
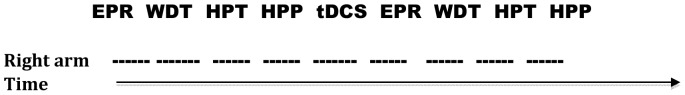
Timing of trial procedures. EPR = electrical pain ratings, WDT = warm detection threshold, HPT = heat pain threshold, rHP = repetetive heat pain, TMS = transcranial magnetic stimulation, tDCS = transcranial direct current stimulation.

Immediately before and after the stimulation, elements of quantitative sensory testing were performed in all patients (thermal perception and pain thresholds). Consecutively, ratings to painful electrical stimuli and to an established repetitive heat pain paradigm were recorded (Figure 2: Trial Procedures). Furthermore, patients were asked after each stimulation session whether they had perceived a subjective change in intensity or quality of their back pain. In order to differentiate ongoing chronic back pain and experimental pain paradigms, the experimental pain was applied to the hand and forearm of patients.

#### Electrical pain

Pain thresholds to an electrical stimulus were determined after placing a custom-build concentric electrode [28] onto the dorsum of the patient's right hand and increasing the intensity of the stimulus until the patient perceived a pricking pain with an intensity of 6/10 on a numerical rating scale (NRS, 0 = no pain, 10 = worst imaginable pain). With this intensity, six trains of painful stimuli were delivered. After each train the participant rated the pain on the NRS. The mean perceived pain intensity was calculated from all 6 trains (EPR). The electrical stimuli (duration 6 msec, frequency 200 Hz, pulse width 500 msec) were generated using a constant current stimulator (Digitimer DS7A, Letchworth Garden City, UK).

#### Thermal pain

Thermal thresholds were determined by using a TSA-II (Medoc Ltd. Advanced Medical Systems, Ramat Yishai, Israel) device with a thermode of Peltier elements (contact area 16×16 mm, 32°C baseline temperature, ramped stimuli with 1°C/s). The thermode was placed on the participant's right forearm. Initially, warm detection thresholds (WDT) were measured, followed by heat pain thresholds (HPT). The mean threshold temperature of three measurements was calculated.

Consecutively, a suprathreshold heat pain paradigm that had been used in a number of previous investigations [29], [30] was applied. The paradigm consisted of 10 blocks with 6 noxious heat stimuli each (baseline temperature 32.0°C, target temperature 48.0°C for 6 sec; temperature rise 10°C/sec). Heat stimuli were triggered every 15 sec by an external computer with a custom-written application (Presentation 11, Neurobehavioral Systems Inc., Albany, CA, USA). After each block, subjects were asked to rate perceived pain on a visual analog rating scale (VAS) ranging from 0 (no pain) to 100 (worst imaginable pain) using a computed device and stored for offline analysis. For post intervention measures the thermode was placed approximately 2–3 cm distal of the area of erythema maintained from the preceding tests to avoid altered pain ratings due to local sensitization.

### Interventions

#### Single-pulse transcranial magnetic stimulation (TMS)

The stimulation site for subsequent tDCS was determined by identifying the motor cortical representational fields of the right abductor digiti minimi muscle by single pulse TMS to the left motor cortex using a magnetic stimulator (Magstim 200, The Magstim Company, Dyfed, UK) and a figure-of-eight shaped magnetic coil with an outer diameter of 70 mm. The corresponding spot was marked with a felt tip pen.

#### Transcranial direct current stimulation (tDCS)

The direct current stimulation was delivered by a battery driven device (DC-Stimulator, neuroConn, Ilmenau, Germany) and applied via saline-soaked sponge electrodes sized 5×7 cm (35 cm^2^) consequently resulting in a current density of 0.029 mA/cm^2^ over the left motor cortex. The stimulation paradigm was chosen according to the most frequently applied tDCS paradigm in previous research on experimental pain [5], [8], [9]: The stimulation lasted for 15 minutes with an intensity of 1 mA in a single session. The reference electrode was positioned above the right orbita. The electrodes were securely attached to the site of stimulation with an elastic bandage. Participants were asked to remain quiet in a supine position during stimulation.

For sham stimulation the device switched off automatically after a 30 second period of stimulation that mimicked the tingling or mild burning sensation commonly perceived by the participants. This brief period of stimulation does not result in any neurophysiological changes [31]. The device display continued to indicate the stimulation time, independent whether verum or sham stimulation was delivered. Whether the stimulator delivered verum or sham stimulation was determined by a five digit code thereby allowing a blinding of patient and assessor. Previous research has shown that this type of blinding is effective at low stimulation intensities [32]. To identify whether the blinding of the participant was successful, the participant was asked afterwards whether he/she believed to have received an active or a sham intervention.

Single sessions were separated by at least one week [33] to avoid carry-over effects.

### Analysis of the data

To analyze the response of each individual patient, percentage of electrical and thermal pain intensity change was calculated pre and post anodal and cathodal stimulation. If a patient rated the perceived pain intensity more than 30% higher or lower than baseline, this was regarded as a response. As there are no recommendations for the evaluation of provoked pain in patients with chronic pain, a cut-off level of 30% was chosen according to minimal important change recommendations for patients with low back pain [34].

To test for distribution differences, Fisher's Exact probability test was applied with the Freeman-Halton extension as sample sizes in particular cells were small with expected frequencies below 5 [35]. The analysis was repeated with a more conservative cut-off of 50% in case of significant results of distribution frequencies. Results are given as two-tailed probability values.

As distribution was not normal in a subset of variables, all data were transformed into the decadic logarithm to achieve a secondary normal distribution before further parametric analysis by means of analysis of variance for repeated measures and paired t-tests. A small constant (0.1) was added to avoid loss of zero-values (for theoretical background see [36], [37]).

The group analysis of pain ratings to repetitive thermal pain was conducted with raw data and additionally with normalized data as the first pain rating differed substantially across all participants. Normalization was achieved by subtracting the second and all subsequent ratings from the first rating for each patient. Arithmetic mean heat pain ratings were calculated across the 10 respectively 9 blocks of heat pain stimulation. Mean ratings to repetitive heat pain were then analyzed separately by a two-way analysis of variance for repeated measures (ANOVA) with the factors stimulation mode (STIM; anodal, cathodal, sham) and time with respect to the intervention (PREPOST; before and after tDCS intervention). Additionally, a repeated measures ANOVA was calculated with pain ratings of the 10 successive blocks each with the factors stimulation mode (STIM; anodal, cathodal, sham), time with respect to the intervention (PREPOST; before and after tDCS intervention) and pain rating per block (BLOCK; pain ratings to heat pain paradigm block 1–10 respectively 1–9 for normalized data).

Changes of mean electrical and thermal pain ratings, WDT and HPT were analyzed separately by a two-way analysis of variance for repeated measures (ANOVA) with the factors stimulation mode (STIM; anodal, cathodal, sham) and time with respect to the intervention (PREPOST; before and after tDCS intervention). Uncorrected paired t-tests were used in all ANOVAs as post-hoc tests in case of significant main effects or interactions.

As normal distribution is difficult to ascertain in small samples, the analysis was repeated with non-parametric tests (Friedman's ANOVA and Wilcoxon test). In case of repeated measures ANOVAs, pre-post differences were calculated and inserted into Friedman's ANOVA for the factor STIM (anodal, cathodal, sham). Additionally, all pre and all post results were also inserted into Friedman's ANOVA. Pre-post differences were examined by means of the Wilcoxon test. Elements of the 3 factorial repeated measures ANOVA for all consecutive blocks of repetitive heat pain were not repeated if results were non-significant and results of the non-parametric testing with mean pain ratings for all 10 blocks of repetitive heat pain were also non-significant.

Rate of correct blinding was tested as a 2×2 contingency table for statistical significance with the Fisher's exact test. P values<.05 were considered significant. Statistical analysis was performed with SPSS 17 for Windows (SPSS Inc., Chicago, Illinois, USA).

Additionally, Pearson's correlation coefficient was calculated for baseline ratings of clinical pain and first electrical and thermal pain ratings and heat pain thresholds recorded immediately thereafter.

## Results

### Descriptive statistics

A total of 15 subjects with chronic low back pain were recruited. One subject (s04) dropped out before completion of the study, because she was unable to tolerate the repetitive heat pain. The remaining 14 subjects (6 male, 8 female) were included into the statistical analysis. An overview of baseline characteristics is given in Table 1. Preventative medication was taken by 5 patients (s01: aceclofenac, s06: flupirtin, s10: pregabalin and amitriptylin, s12: mirtazapine and s15: etoricoxibe). The dosage was kept constant throughout the study period, no further acute medication was taken in the preceding 24 hours.

**Table 1:** Patient characteristics at baseline.

subject	gender	age	duration of BP (years)	average pain in the past 24 hrs NRS 0-10	painful days (within the last 31 days)	HADS		R MD Q
						anxiety	depression	
**s01**	m	70	13	8	31	9	4	7
**s02**	f	69	6	2	10	2	1	7
**s03**	f	52	6	4	31	7	4	4
**s04**	f	49	15	6	31	8	4	5
**s05**	m	33	3	4	31	7	5	5
**s06**	f	41	23	5	31	8	7	6
**s07**	m	57	20	7	31	12	11	7
**s08**	f	53	3	5	31	11	9	13
**s09**	f	48	0.5	2	31	9	11	12
**s10**	f	50	4	4	31	11	12	9
**s11**	m	67	19	1	31	1	2	10
**s12**	f	35	6	1,5	31	7	4	7
**s13**	m	38	8	5	31	2	5	-
**s14**	m	39	22	5	28	7	5	4
**s15**	f	30	20	3	10	8	6	6
**mean**	**male∶female = 1∶1.5**	**48.7**	**11.2**	**4.3**	**28**	**7.2**	**6**	**7.3**

BP = back pain, NRS = numerical rating scale, HADS = hospital anxiety and depression scale, RMDQ = Roland-Morris-Disability Questionnaire.

### Responses to electrical pain

There was no significant difference of the perceived mean pain intensity before and after tDCS stimulation for the factors time (PREPOST: F(1,13) = 0.85; p = 0.374) and stimulation modality (STIM: F(2,26) = 0.93; p = 0.379) and no interaction between STIM*PREPOST (F(2,26) = 1.18; p = 0.323). Non-parametric testing did not differ from these findings. Mean electrical pain ratings are shown in Table 2.

**Table 2:** Mean electrical pain (EPR) on a 0-100 numerical rating scale, warm detection thresholds (WDT) and heat pain thresholds (HPT) (all non-normalised) before (pre) and after (post) application of anodal, cathodal and sham tDCS.

		EPR	WDT	HPT
**Cathodal**	**pre**	57.1	37.3°C	44.9°C
	**post**	57.9	37.8°C	44.8°C
**Anodal**	**pre**	54.5	37.4°C	44.9°C
	**post**	58.7	38.1°C	45.9°C
**Sham**	**pre**	61.4	37.3°C	45.0°C
	**post**	59.7	38.4°C	44.8°C

Individual patient responses are shown in Table 3. There were no significant distribution differences for the 30% cut-off (P = 0.093).

**Table 3:** Percentage of change in electrical and thermal pain ratings in individual patients.

Heat pain									
patient no.	anodal pre	anodal post	% change	cathodal pre	cathodal post	% change	sham pre	sham post	% change
s01_02	67,90	70,90	4	65,80	70,80	8	81,10	70,30	−13
s02_02	44,00	45,30	3	45,50			28,10	29,60	5
s03_01	50,50	21,40	−58	34,30	24,80	−28	44,20	55,30	25
s05_03	59,56	56,80	−5	58,20	62,70	8	63,50	68,30	8
s06_03	11,60	12,90	11	48,40	51,40	6	42,90		
s07_03	8,70	8,00	−8	12,20	10,00	−18	14,80	17,22	16
s08_01	37,10	28,40	−23	38,30	34,60	−10	47,30	33,90	−28
s09_02	88,70	86,80	−2	85,60	89,70	5	93,20	95,00	2
s10_02	78,10	78,10	0	79,10	91,10	15	69,70	81,80	17
s11_03	34,70	37,30	7	34,33	38,30	12	32,20	38,10	18
s12_01	78,50	81,30	4	74,20	68,20	−8	44,50	28,80	−35
s13_02	47,40	53,90	14	36,80	35,20	−4	60,00	41,70	−31
s14_03	68,80	67,40	−2	76,00	75,00	−1	85,10	74,50	−12
s15_01	99,80	98,80	−1	96,00	98,50	3	98,60	96,90	−2
mean	55,38	53,38	−3,97	56,05	57,72	−1,04	57,51	56,26	−2,28
**Electrical pain**									
s01_02	33,50	58,67	75	70,00	71,67	2	39,17	45,67	17
s02-02	82,00	79,67	−3	51,50	49,00	−5	88,00	76,50	−13
s03_01	42,33	27,50	−35	20,00	15,17	−24	46,17	55,67	21
s04_01				74,50	58,67	−21			
s05_03	63,17	66,50	5	69,50	75,50	9	74,67	76,50	2
s06_03	44,83	55,50	24	50,67	47,33	−7	53,83	51,33	−5
s07_03	25,67	38,83	51	29,00	38,83	34	51,67	43,50	−16
s08_01	51,83	43,17	−17	68,00	60,50	−11	67,83	57,00	−16
s09_02	88,67	94,83	7	81,67	91,00	11	93,50	97,00	4
s10_02	75,17	65,33	−13	64,17	72,00	12	69,17	66,33	−4
s11_03	38,33	54,50	42	31,33	48,83	56	48,33	47,67	−1
s12_01	56,17	57,00	1	56,33	50,67	−10	45,50	39,83	−12
s13_02	34,50	35,33	2	37,67	35,33	−6	30,83	38,50	25
s14_03	71,67	74,83	4	72,83	82,17	13	71,00	64,83	−9
s15_01	55,17	70,67	28	78,67	71,67	−9	80,33	75,17	−6
mean	54,50	58,74	12,38	57,06	57,89	2,95	61,43	59,68	−0,99

Changes of >30% are highlighted.

### Thermal thresholds

Due to technical problems during data acquisition (intermittent thermode malfunction), data of 2 patients could not be collected (S02 for cathodal and S06 for sham stimulation). Thermal threshold and pain evaluation is therefore based on 11 respectively 12 complete sets of data respectively.

Mean values for thermal thresholds are given in Table 2. For WDT, a repeated measures ANOVA yielded significant results for the factor PREPOST (F(1,10) = 6.6; p = 0.028), while STIM was not significant (F(2,20) = 0.98; p = 0.393). The interaction between the two factors was significant (STIM*PREPOST: F(2,20) = 3.84; p = 0.039) and *post hoc* t-tests showed that this effect was driven by the comparison of anodal results before and after stimulation (t(13) = −2.72; p = 0.018). Non-parametric tests confirmed these findings.

There were no significant differences of HPT before and after tDCS (PREPOST: F(1,9) = 0.82; p = 0.389) and between treatment modalities (STIM: F(2,18) = 1.27; p = 0.306). No interaction between the two factors (STIM*PREPOST: F(2,18) = 1.44; p = 0.263) could be found. Non-parametric testing did not differ from parametric testing.

### Repetitive heat pain stimulation

Mean baseline pain ratings (rating of initial block of experimental pain, data not shown) did not show any significant *a priori* difference (STIM: F(2,26) = 1.14; p = 0.319) between the three experimental sessions in a one way ANOVA. No significant difference of *mean* heat pain intensity over all 10 blocks before and after tDCS (PREPOST: F(1,11) = 2.09; p = 0.176) and between stimulation modalities (STIM: F(2,22) = 0.47; p = 0.555) could be found (Table 4). In addition, there was no significant interaction (STIM*PREPOST: F(2,22) = 0.26; p = 0.652).

**Table 4:** Mean ratings to repetitive heat pain (non-normalised) before (pre) and after (post) application of anodal, cathodal and sham tDCS on a visual analog scale (0-100).

		Rating 1	Rating 2	Rating 3	Rating 4	Rating 5	Rating 6	Rating 7	Rating 8	Rating 9	Rating 10	Mean
**Cathodal**	*pre*	52.14	51.43	53.93	53.21	57.29	58.36	57.71	57	59.14	60.31	**56.05**
	*post*	56.46	57.38	58.92	59.46	58.15	60.08	57.23	57.85	56.77	54.85	**57.72**
**Anodal**	*pre*	50.18	53.07	54.29	55.14	56.07	56.64	57.43	57.57	55.86	57.57	**55.38**
	*post*	53.36	49.86	50.21	52.21	53.5	55.14	54.64	54.86	55.14	54.86	**53.38**
**Sham**	*pre*	49.64	53.57	54	55.93	57.64	58.79	60.79	59.71	62.29	62.79	**57.51**
	*post*	52.69	51	54.31	55.85	57	57.08	57.77	59.15	58.92	58.86	**56.26**

Cumulative ratings are given for each of the ten blocks consisting of 6 brief nociceptive stimuli each.

Non-parametric testing did not differ from these findings.

Likewise, a three factor repeated measures ANOVA using non-normalized data across all 10 blocks did not yield significant results for the factors PREPOST: F(1,11) = 1.67; p = 0.222) STIM (F(2,22) = 0.56; p = 0.509) and BLOCK (F(9,99) = 2.87; p = 0.103) and the corresponding interactions (STIM*PREPOST: F(2,22) = 0.13; p = 0.767; STIM*BLOCK: F(18,198) = 0.70; p = 0.554; PREPOST*BLOCK: F(9,99) = 1.86; p = 0.188; STIM*PREPOST*BLOCK: F(18,198) = 1.1; p = 0.363). When repeated with normalized data, results were comparable.

Individual patient responses are shown in Table 3. There were no significant distribution differences (P = 0.762) for a 30% cut-off.

### Perceived change in back pain

All patients reported that their back pain remained unchanged in intensity and quality after each stimulation session compared to baseline assessment as given in Table 1.

### Correlation between clinical pain and experimental pain

Pearson's correlation coefficient shows no relevant correlation between baseline ratings of clinical pain and first ratings of electrical and thermal pain and heat pain thresholds recorded immediately thereafter (r≤0.09 for all variables except for first rating of repetitive heat pain with r = −0.208; p≥0.475 for all variables).

### Blinding

Blinding was successful as confirmed by a non-significant result in Fisher's Exact test (p = 0.45).

## Discussion

A single 15 minutes session of anodal or cathodal stimulation at 1 mA over the motor cortex did not significantly alter the perception of noxious thermal and electrical stimuli and thermal pain thresholds applied to the right hand in patients with chronic low back pain. The analysis according to responders and non-responders did not show significant distribution differences across the stimulation modes. Neither cathodal nor anodal or sham stimulation influenced the perceived intensity or quality of the patients' back pain. However, warm detection thresholds were significantly elevated after anodal tDCS.

A change in clinical pain was not expected after a single 15 minutes session of tDCS with an intensity of 1 mA and clinical pain was not the main focus of this trial. A pain reducing effect on chronic pain has already been demonstrated by other authors who all applied more than one session of tDCS and commonly used longer durations (20 minutes) and higher intensities (2 mA) [11], [12], [13], [38], [39]. However, experimental pain was modulated by tDCS mostly with stimulation intensities of 1 mA in other studies [5], [6], [7], [8], [9].

All patients stated that they could clearly distinguish between clinical and experimental pain. In addition, there was no relevant correlation between baseline clinical pain and first ratings of experimental thermal and electrical pain. Therefore, it is unlikely that an attentional deficit lead to insufficient discrimination between clinical and experimental pain.

### Modulation of thermal detection thresholds

The only significant finding was an increase in warm detection thresholds after anodal tDCS. Thermal sensation may be more susceptible to cortical excitability changes following tDCS than nociception, especially as tDCS of the primary motor cortex induces widespread central activity changes [2]. In addition, cortical thickness in different cortical areas correlates with warm detection and heat pain thresholds [40]. However, it has been shown that thermal perception and nociception have their own labeled lines and receptors resulting in a central integration of all neuronal input [41]. Likewise, Bachmann et al did not report a change in WDT following tDCS in healthy participants [8]. Therefore, our findings should be interpreted with caution: WDT were increased both after cathodal and anodal stimulation in our sample. As this is contrary to a concept of diametrically opposed neurophysiological effects of anodal and cathodal stimulation on cortical excitability [1], our findings are difficult to explain and may be due to unspecific effects such as a decline of attention during the sessions – an effect also observed in the study of Lefaucheur and colleagues [25]. The fact that we examined statistically significant main effects and interactions by uncorrected *post hoc* tests supports a careful appraisal of these findings.

### tDCS in clinical and experimental pain

Our results did not reproduce the effect reported by publications on tDCS for the reduction of experimental pain in healthy participants. Potential explanations for the different response of chronic pain patients to experimental pain compared to healthy participants as reported in previous publications [5], [6], [7], [8], [9] could be the structural and functional changes of the pain processing system known to occur in longstanding painful conditions. Structural changes in chronic low back pain patients include a typical pattern of grey matter loss in regions associated with pain processing, such as the cingulate cortex, insula and orbito-frontal cortex [42], [43], [44]. These regions function as multi-integrative structures during the experience and the anticipation of pain [30].

Functional adaptations in chronic pain patients comprise altered cortical activity and changes of the cortical representational fields of the affected body parts [45], [46], [47]. It is therefore conceivable that the efficacy of tDCS differs between chronic pain patients and healthy volunteers. One could argue that the intensity, frequency and duration of the tDCS stimulation may not have been sufficient to challenge the structurally and functionally adapted brain of chronic pain patients. While trials on healthy participants did report pain perception changes after a single session of stimulation with 1 mA [5], [7], [8], [9], trials on chronic pain populations mostly applied higher intensities of tDCS, typically 2 mA for 20 minutes on 2–10 consecutive days [11], [12], [13], [38], [39]. The stimulation paradigm in this study (1 mA, 15 minutes, single session) was chosen, because it was the paradigm most frequently used in studies on experimental pain [5], [8], [9] and has been advocated to be more reliable in blinding the participants towards the stimulation mode [13]. However, an in depth analysis of previous publications showed that the effects of tDCS on experimental pain ratings induced by laser and electrical stimuli were small or absent. Effects on components of evoked potentials were more consistent [7], [9], [48] suggesting divergent effects on psychophysical and neurophysiological outcome parameters.

The role of the motor cortex in neuromodulation may have been overestimated as effects of motor cortex stimulation have been shown to be conveyed by a network of other interconnected brain areas [49]. Recent research in monkeys has shown that noxious heat stimuli result in a weaker response of the motor cortex in comparison to other brain regions, such as the somatosensory cortex [50]. Based on the findings of Antal et al [5] the somatosensory cortex could have been a better target for tDCS. However, therapeutic effects in patients with chronic clinical pain have primarily been reported after motor cortex stimulation for the treatment of chronic pain [51], [52].

### Comparison to other non-invasive neuromodulatory approaches

These results differ from those reported in two other studies on the effects of repetitive transcranial magnetic stimulation (rTMS) on experimental pain in patients suffering from a chronic pain condition [25], [26]. rTMS is a different type of non-invasive brain stimulation that uses a transient magnetic field to elicit electrical currents in the stimulated area that - not unlike tDCS- modulate cortical excitability and subsequently in a top-down order a network of structures crucial to nociception [53]. The differences between both techniques have been discussed in detail elsewhere [54] with one of the main differences being that TMS results in more focal effects. This may potentially explain why a single session of high frequency rTMS is sufficient to alter experimental pain even in patients with chronic pain while this effect could not be reproduced with tDCS.

### Technical considerations and limitations

We can exclude methodological shortcomings which could explain a missing effect for the following reasons: as previously shown, effects of tDCS on nociception persist for a maximum of 150 minutes after tDCS [55]. As the three stimulation dates were separated by a minimum of 5 days, a carry-over effect can be excluded. In addition, the motor cortex was located by means of single-pulse TMS rather than relying on the 10/20 electrode system. In addition, technical details render the motor cortex the target of choice as the exact electrode position can be located by single-pulse TMS.

A larger sample size may have detected an effect that was missed in this study. Chronic low back pain patients are a heterogeneous sample regarding pain presentation and co-morbidities, thereby potentially influencing the effect of an intervention. In addition, the concomitant intake of analgesics antidepressant/antiepileptic drugs in a subgroup of the patients could have influenced our results, although medication was kept constant throughout the study. The participant (s10) who took pregabalin and amitriptylin did not react differently to tDCS than any other participant.

### Conclusion

Single-session anodal and cathodal direct current stimulation did not modulate the perception of nociceptive experimental stimuli in patients with chronic low back pain. Further research is needed to investigate the effect of higher intensities and repetitive application of tDCS as well as stimulation sites other than the motor cortex.
